# Uncertainties in outcome modelling in radiation oncology

**DOI:** 10.1016/j.phro.2025.100774

**Published:** 2025-05-07

**Authors:** Lukas Dünger, Emily Mäusel, Alex Zwanenburg, Steffen Löck

**Affiliations:** a OncoRay – National Center for Radiation Research in Oncology, Faculty of Medicine and University Hospital Carl Gustav Carus, TUD Dresden University of Technology, Helmholtz-Zentrum Dresden-Rossendorf, Dresden, Germany; b National Center for Tumor Diseases (NCT), NCT/UCC Dresden, a partnership between DKFZ, Faculty of Medicine and University Hospital Carl Gustav Carus, TUD Dresden University of Technology, and Helmholtz-Zentrum Dresden-Rossendorf (HZDR), Germany; c German Cancer Consortium (DKTK), Partner Site Dresden, and German Cancer Research Center (DKFZ), Heidelberg, Germany; d Department of Radiotherapy and Radiation Oncology, Faculty of Medicine and University Hospital Carl Gustav Carus, TUD Dresden University of Technology, Dresden, Germany

**Keywords:** Radiation oncology, Outcome, Statistical analyses, Uncertainty modelling

## Abstract

Outcome models predicting e.g. survival, tumour control or radiation-induced toxicities play an important role in the field of radiation oncology. These models aim to support the clinical decision making and pave the way towards personalised treatment. Both validity and reliability of their output are required to facilitate clinical integration. However, models are influenced by uncertainties, arising from data used for model development and model parameters, among others. Therefore, quantifying model uncertainties and addressing their causes promotes the creation of models that are sufficiently reliable for clinical use. This topical review aims to summarise different types and possible sources of uncertainties, presents uncertainty quantification methods applicable to various modelling approaches, and highlights central challenges that need to be addressed in the future.

## Highlights

• Outcome models enable personalised treatment by, e.g. predicting tumour control.
• Predictions may be certain or uncertain, indicating if they should be relied upon.
• This topical review addresses causes and quantification methods for uncertainty.
• High-quality, comprehensive and bias-free data decrease uncertainty.
• Use of uncertainty quantification methods may facilitate clinical translation.

## Graphical abstract

**Figure:**
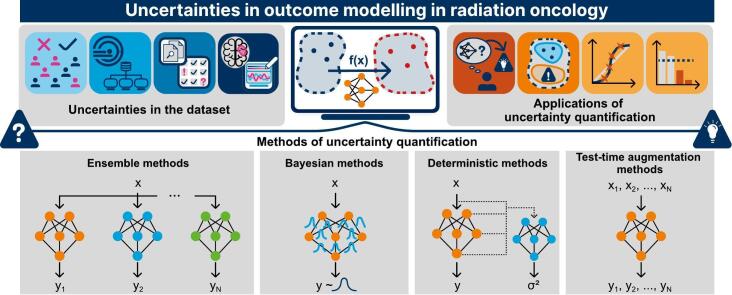

## 1 Introduction

In radiation oncology, models are used to replicate the complex effects of treatment [1]. Such outcome models range from relatively simple models for predicting normal tissue complication probability (NTCP) that are in actual clinical use, e.g. as summarised in the QUANTEC articles [2], to complex artificial-intelligence- (AI) based, multi-modal approaches for treatment personalisation that have gained considerable importance recently [[3], [4], [5], [6], [7], [8], [9], [10], [11], [12]].

Any model is based on observations, i.e. a dataset with observed input and its associated observed outcome. Typical examples of input data are clinical scores and variables, medical imaging, dose plans as well as genetic markers [1]. Common outcomes are radiation toxicities and proxies for tumour control probability (TCP), such as loco-regional control or survival. To create a model, data are first acquired and stored. Subsequently, data are curated, annotated and processed, e.g. by applying normalisation strategies for medical images from different centres, or extracting features as dose-volume histogram parameters. Additionally, modelling assumptions and definitions, such as the selection of a model architecture, the presumption of underlying statistical correlations as well as the choice of relevant input parameters are specified. The model parameters are optimised in a training process to approximate the relationship between the processed input data and the observed outcomes. Finally, independent validation is performed for assessing model generalisability.

Regardless of its complexity, uncertainties are part of every constructed model. They arise from characteristics and limitations in the underlying dataset, modelling assumptions or simplifications as well as from the optimisation process itself [12,13]. Such uncertainties propagate throughout the modelling process and translate into an uncertainty in the outcome predicted by the model. The purpose of outcome models is to support and guide clinical decision making, e.g. for selecting patients that have a relevant beneficial use of a specific treatment strategy [[14], [15], [16]]. To ensure that the expected benefit for patients using outcome models can be realised in clinical practice, uncertainties that might have a relevant effect on the model prediction should be minimised whenever possible, and transparently quantified. Additionally, reliability, interpretability, explainability and the assessment of limitations are of major importance for clinical translation of outcomes models [[3], [4], [5], [6], [7], [8],^11^,^13^]. These issues can be addressed through a comprehensive uncertainty analysis, as shown for medical image analysis [17].

This review aims to provide an overview of sources of uncertainties and quantification strategies for outcome modelling in radiation oncology. We first introduce different types of uncertainty. Focusing on the data collection process, we then discuss uncertainties that are related to the dataset used for modelling. Additionally, we summarise several methods for assessing and quantifying uncertainty before discussing major challenges and central steps that need to be addressed in the future. While we highlight specific approaches that may be useful for outcome modelling in radiation oncology, we do not intend to cover all available methods in the field.

## 2 Defining uncertainty in outcome modelling

**Fig. 1:**
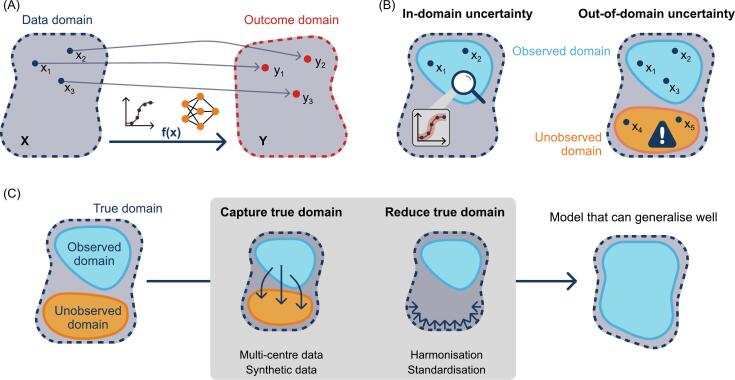
Concept of data domains for addressing different types of uncertainties. (A) During the training process a mapping f(x) between the data points in the data domain x_i_ ∈ X and the outcome domain y_i_ ∈ Y is created, e.g. using logistic regression or a neural network. (B) When applying a model to unseen data, in-domain uncertainty refers to uncertainties for data points which are in the observed domain and have characteristics similar to the training data. In contrast, applying the model to data whose characteristics differ from the samples of the observed domain can lead to out-of-domain uncertainty. (C) In order to achieve a model that can generalise well, capturing and reducing the true domain are two main strategies for reducing uncertainties.

An outcome model in radiation oncology is designed to abstract and replicate the complex treatment response as a functional relationship by conceptually mapping data points from a data domain to an outcome domain, for example using logistic regression or neural networks, see Fig. 1 (A). In the data domain, every point represents data related to a single observation or instance, e.g. for one patient. The outcome domain comprises the associated outcomes, e.g. the grade of a certain side effect for each patient. The input data comprise data points seen by the model in the training or tuning process. They define the observed domain and contain data after acquisition, storage, curation, annotation and processing. Therefore, the reliability of the input data, containing accumulated uncertainty due to the prior processing steps, is of central importance. Possible sources of errors and uncertainties will be further addressed in Section 3.

In practice, models are intended to predict an outcome for new, previously unseen data. Errors or unreliable predictions may occur for different reasons, e.g. due to an inadequate functional mapping or if there is a mismatch in the input or outcome domain between the training and the new dataset. Using the concept of data domains, three different types of uncertainty can be differentiated [18]: (i) In-domain uncertainty [19] is the uncertainty of a prediction for data that are assumed to have similar characteristics as the training data, i.e. data that are in the observed domain, see Fig. 1 (B). (ii) Out-of-domain uncertainty [20] comprises uncertainty of the model applied to data that are outside the observed domain, e.g. when applying a model trained on magnetic resonance (MR) images to computed tomography (CT) images. Such data are subject to a high degree of uncertainty and predictions may not be reliable, see Fig. 1 (B). (iii) Domain-shift uncertainty [21] − a subcategory of out-of-domain uncertainty − arises from a population-wide shift in characteristics compared to the observed domain. This can be a relevant problem in the medical field, as data are acquired at institutions with individual measurement and imaging devices, patient populations, physicians, and operating procedures. Moreover, shifts can also occur over time, as technology progresses and treatment improves. For example, a hospital might introduce a new MR imaging protocol for T2-weighted brain scans, resulting in a previously trained model no longer working as expected in patients imaged using the new protocol.

**Fig. 2:**
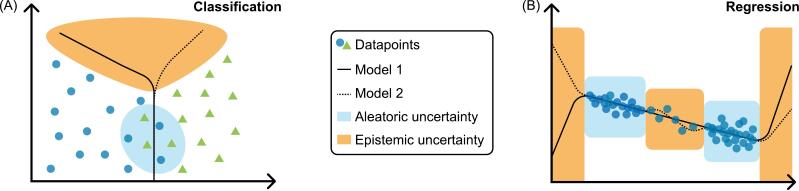
Aleatoric and epistemic uncertainty. Models 1 and 2 are trained using the same data points, but their predictions are influenced by aleatoric and epistemic uncertainties. For a classification task (A), the aleatoric uncertainty is high in the domain with overlapping data points due to inherent randomness of the data, whereas the epistemic uncertainty is high in areas with missing data points. Similarly, for a regression task (B), the epistemic uncertainty is high in areas with no or only sparse data points. In areas with numerous data points, inherent randomness results in a high aleatoric uncertainty.

From another perspective, two main types of uncertainty can be distinguished, namely aleatoric and epistemic uncertainty [[22], [23], [24]]. This classification allows for differentiating between underlying mechanisms of uncertainty. Aleatoric uncertainty is related to noise and randomness in the data and is also called stochastic uncertainty. In the data domain, aleatoric uncertainty is observed through spatial overlap between instances with different outcome and spatial spread between instances with the same outcome. A simplified illustration for a classification and a regression task is shown in Fig. 2 (A) and (B), respectively. Epistemic uncertainty is caused by a lack of knowledge, e.g. about perfect model parameters or by sparsity of observations. In the data domain, as illustrated in Fig. 2, epistemic uncertainty is observed where instances are sparse or missing altogether. Epistemic uncertainty can be considered as reducible, e.g. by including additional patients, simplifying the domain by reducing the number of variables, or using less complex model architectures to simplify the mapping between input and output domains.

## 3 Reliability of input data

**Fig. 3:**
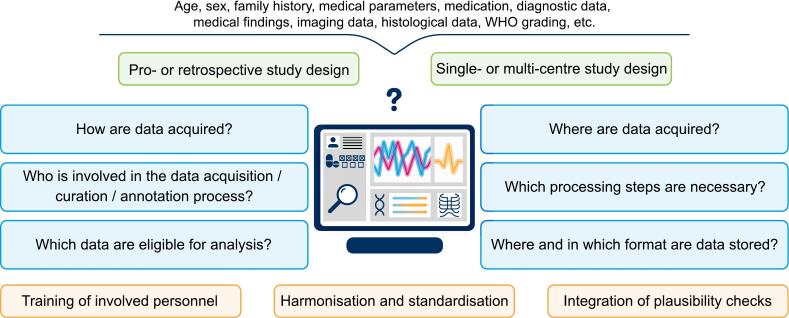
Important elements of the data collection process. Main study designs (top, green), central questions related to data collection requirements (middle, blue) as well as relevant strategies (bottom, orange) that can be used to create a reliable dataset are shown. (For interpretation of the references to colour in this figure legend, the reader is referred to the web version of this article.)

Models require data. Thus, the data collection process is of central importance. A data analysis plan or structured study protocol systematically details and addresses key questions and decisions (see Fig. 3) and helps to identify, assess and reduce errors and uncertainties. Below, we describe the main elements of a data analysis plan. Subsequently, we summarise potential issues contributing to the overall uncertainty in a dataset and outline possible strategies for reducing them.

### 3.1 Data analysis plan

**Fig. 4:**
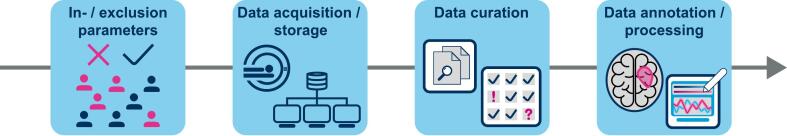
Data analysis plan. A data analysis plan contains several steps that need to be addressed during the creation of a dataset for outcome modelling. The individual steps build on each other: First, in- and exclusion parameters are needed, defining the characteristics of the cohort. Second, protocols, parameters and data storage for data acquisition must be defined. Third, methods for data curation including analysis, sorting and validation of the content must be outlined. Fourth, data annotation and processing steps need to be defined, e.g. for segmentation or feature selection.

A data analysis plan details the processing steps of a study from the definition of inclusion parameters to the compilation of the final dataset. As illustrated in Fig. 4, four main steps can be defined:

(i) The inclusion and exclusion parameters detail which patients are eligible for training the model and thus define the data domains. A (retrospective) patient cohort could be formed by patients with a certain diagnosis who received a specific treatment and have complete data. Exclusion criteria could be previous treatment with radiotherapy or noticeable artefacts in image data. The sample size should also be considered, as an insufficient sample size leads to reduced power, i.e. increases the probability of a false negative study result. Moreover, with small sample sizes the risk of overfitting increases. Since data acquisition and curation can be cumbersome and expensive, sample size analyses with realistic estimates for the expected effect can be performed before data collection. For regression-based prediction models there is guidance for sample size estimation [[25], [26], [27], [28]]. However, for machine- and deep-learning-based models, there is an overall scarcity of concepts to estimate a sufficient sample size pre-hoc, e.g. as shown for medical imaging analysis [29]. Estimating sample size based on rules of thumbs, e.g. ten events per variable [[30], [31], [32]], might be inadequate and should be study and context dependent [25,[33], [34], [35]]. A lower bound of an appropriate number of samples can be estimated based on available concepts for regression-based approaches [36]. For particular machine learning algorithms, model-based methods can be used for a pre-hoc estimation of an adequate sample size [29]. A subsequent verification and post-hoc validation of the estimated sample size can be conducted by assessing model performance, for example by using subsampling and cross-validation [29] or by evaluating the effect size and accuracy [37].

(ii) Data acquisition and storage are outlined. The plan details which clinical parameters are considered, how and at which time point they were recorded, and how and where the relevant data should be stored. The image acquisition protocols as well as the radiotherapy planning method are described. Typically, medical images and dose distributions are stored as DICOM files in a picture archiving and communication system (PACS). Clinical parameters and scoring are available from the electronic health record (EHR) or a radiotherapy or oncology information system.

(iii) The data analysis plan describes what curation is required to ensure overall consistency in the dataset. Plausibility and validity of clinical and treatment parameters need to be verified and medical images should be checked for artefacts, corrupted or incorrectly assigned DICOM files, etc.

(iv) Data annotation and processing requirements are often induced by the model choice. For instance, models might have a fixed input size, and imaging and dose data need to be processed to conform to those requirements. Furthermore, annotation and processing steps of the data are detailed, e.g. normalisation and interpolation strategies, to enable a consistent voxel size and spatial orientation of the imaging data.

### 3.2 Errors and uncertainties in datasets

The data analysis plan presented in Section 3.1 gives rise to potential issues contributing to an overall uncertainty. Firstly, clinical parameters often rely on manual input [38], which can have outright errors as typos. Uncertainty can also be introduced when data needs to be interpreted [[38], [39], [40]] or when information is lost at the transmission between the patients, clinicians, and researchers [41]. Here, the reliability of all involved data contributors is of major importance [41,42].

Secondly, data are subject to inherent variability, i.e. aleatoric uncertainty. Examples include: (i) heterogeneity in the response to treatment between individual patients or tumours [43] and (ii) variability in medical imaging, e.g. differences in the accumulation and assessment of a PET tracer [44,45]. (iii) Tumour biopsies and the derived information may not represent the whole tumour due to underlying tissue heterogeneity [46,47]. (iv) Data may be sensitive to inter-observer variability impacting radiotherapy planning [48,49], delineations of target volumes and organs-at-risk [50,51], or extracted imaging features [52,53]. (v) Differences between the planned dose distribution and the actual delivered dose may occur, e.g. due to intra-treatment differences by organ movement [54] and inter-treatment variations due to anatomical changes [55]. (vi) Survival endpoints, e.g. local tumour control or progression-free survival, depend on the time, frequency and duration of follow-up, the use of imaging surveillance [56], the chosen imaging modality and technique [57,58] and capacity of the clinician to detect recurrence. (vii) Subjective information, such as patient-reported outcomes, may not be reproducible by objective measures, and affected by phrasing [59] and patient reaction to specific questions [60].

Thirdly, data may be unstructured and difficult to process directly using software. For instance, radiological findings, e.g. containing information about the occurrence of recurrences, may be reported in free text. Thus, digitalisation of written notes and natural language processing [[61], [62], [63], [64]] are important tools to identify structured, machine-readable information. Furthermore, for retrospective studies it may be challenging to obtain reliable data from medical databases and information systems [[65], [66], [67], [68], [69]]. For example, if data in health records are redundant or siloed, i.e. stored within systems that have no interaction, available information can be cluttered, e.g. by excessive copying and pasting [66], or even conflictive [41,70]. Records might also lack consistent identifiers, i.e. resulting in inconsistent DICOM tags [[71], [72], [73], [74]]. Consequently, data require close inspection, or the use of specific tools to assist in identification and selection, e.g. to automatically classify MR imaging data [73,75].

Fourthly, data may be partly missing or incomplete for part of the patients [69,76]. Not or poorly addressing the existence of missing data can increase the overall uncertainty, as e.g. shown for the impact on machine learning classifiers [77]. Consequently, the reason for missing data should be evaluated and appropriate methods should be applied to ensure reliable modelling [76,78,79].

### 3.3 Bias and confounding

Assessment of bias helps to ensure that the final model is generalisable. Bias can be interpreted as a systematic difference causing a tendency towards non-generalisable results [59]. The sources of bias can be highly diverse and might influence all study processes. Selected examples for sources of bias that can affect outcome analysis in the field of radiation oncology are summarised in Table 1.

**Table 1:** Non-exhaustive overview of possible sources of bias for outcome models in radiation oncology.

Source of bias	Examples
Differences between centres	• Access to care and infrastructure [[80], [81], [82], [83], [84]] • Quality and safety considerations [85,86] • Frequency of types of treatment [87]
Differences in patient population	• Disease prevalence between centres [88,89] • Lifestyle patterns, socioeconomic status [90,91] • Genetic predispositions [92]
Differences in treatment protocols	• Dose prescriptions [93,94] • Treatment planning [95] • Patient alignment procedures [96] • Routine clinical practice [97] • Deviations from study protocols [[98], [99], [100]]
Differences in equipment	• MR scanners [101,102] • PET scanners [44,103]
Differences in data acquisition protocols	• Grading of radiotoxicities [104] • Scan settings, as slice thickness [105] • Follow-up examinations [106]
Differences in annotation protocols	• Protocols for delineating tumours / organs-at-risk [107,108]
Differences in ontologies	• Naming conventions for anatomical structures [[109], [110], [111], [112]]
Differences in software and processing parameters	• Image registration [113] • Definition of dose [114] • Size of the dose grid [115] • Dose calculation algorithm [116] • Image processing [117,118]

Additionally, the presence of confounders can negatively impact the performance of an outcome model. Confounders are parameters (observed or hidden) that influence other parameters and the outcome simultaneously, and lead to non-causal associations [59]. For instance, concomitant chemotherapy can be a confounder for the comparison of side effects between photon and proton irradiation when its utilisation differs between these two groups while directly affecting the studied outcome. Confounding variables can be addressed using different statistical techniques. A relevant strategy to diminish the influence of confounding variables in non-randomised studies is matching, e.g. based on the propensity score [119]. Nevertheless, there are limitations of such methods [59] which need to be taken into account.

Moreover, in time-to-event analyses competing events may preclude an event of interest, e.g. patients may die due to comorbidities before a recurrence can be observed. Competing events can lead to biased model output and should be identified and handled using appropriate methods [[120], [121], [122], [123], [124], [125], [126], [127], [128], [129]].

### 3.4 Reducing uncertainties

Both errors and biases give rise to uncertainty and should be reduced or mitigated when possible. Reducing or removing errors requires that their causes are addressed. For example, error rates in EHR systems might be reduced by training of the involved personnel, e.g. to improve medical record abstraction [130]. Integrated quality assurance and (automated) plausibility checks, e.g. for radiation plan quality [131,132], may improve data consistency.

Preventing negative effects of bias is often challenging. The aim is to ensure data acquired elsewhere or prospectively can be associated with the observed domain. This helps a model to generalise to new data and suggests two main strategies: (i) capturing the true data domain that is the union of all data domains (including unseen ones) during training and (ii) reducing the true data domain by reducing or mitigating biases. These strategies are schematically illustrated in Fig. 1 (C).

The first strategy (i) suggests three approaches: (a) to collect data from more patients, preferably from multiple centres to better capture biases related to equipment and patient populations. Additionally, distributed or federated learning [133,134] can be applied to increase the study populations. (b) To use existing patient data to synthesise samples, e.g. through augmentation of medical imaging [135]. (c) To use foundational models that capture the salient characteristics of the true data domain and use these for training in a transfer learning approach, e.g. to discover new imaging biomarkers [136].

The second strategy (ii) revolves around harmonisation and standardisation to reduce and remove biases. Various harmonisation techniques and metrics exist [137,138]. Relevant techniques include cross-calibration of imaging equipment [139], intensity normalisation of imaging data [140] as well as the implementation of standardised protocols, e.g. as proposed by the Image Biomarker Standardization Initiative (IBSI) in the scope of radiomics [141,142]. Additionally, AI-based approaches can be integrated in the data collection process to ensure a high degree of standardisation and harmonisation, e.g. automatic segmentation or treatment planning algorithms allow to address inter-observer or inter-centre variabilities.

A complimentary approach is adjusting the dimensionality of the true data domain. The data domain can comprise numerous candidate variables. Using an inadequate number of variables can lead to under- or overfitting and can consequently increase the overall uncertainty [143]. To address this issue, many different algorithms are available for selecting relevant and non-redundant variables [[144], [145], [146]]. However, under- and overfitting may be less relevant when sufficient data are available, and model algorithms are selected that can operate in non-classical regimes, e.g. random forests or neural networks [147].

In summary, high data quality helps reduce uncertainties. However, measuring data quality is not trivial. Operational definitions and acceptance criteria are necessary [38] and harmonisation frameworks of key-concepts [[148], [149], [150], [151]], e.g. for completeness or accuracy, should be used consistently. Uncertainty reduction – especially in post-hoc and external settings where a model was already developed – requires that data collection methods and characteristics of the dataset are transparently and comprehensively reported [152]. This ensures that biases or domain shifts limiting the applicability of the model can be identified. A data quality summary table [42] or a model card [[13], [153]] can help to transparently report on data. Furthermore, consistency in conducting and reporting analyses, e.g. when reporting on inter-observer variabilities [154], is necessary. Additionally, there is a need for a strict and complete compliance to reporting guidelines as TRIPOD [155] or CONSORT [156] as well as their AI-related extensions TRIPOD + AI [157] and CONSORT-AI [158]. Regarding the datasets, following guidelines as the FAIR principles [159] is of crucial importance to enable transparent, repeatable and reliable research.

## 4 Uncertainty quantification

Every model is influenced by uncertainties. Despite extensive efforts to reduce uncertainty in the dataset (see Section 3.4), model uncertainties are furthermore introduced by simplifications, assumptions and model parameters. For this reason, the quantification of existing uncertainty is of crucial importance. Various methods allow for assessing uncertainty of model predictions. Below we focus on the direct quantification of uncertainty in model predictions and assess its effect on model reliability. An example demonstrating the implementation of selected concepts for a logistic regression model to predict a binary outcome is provided as a Jupyter Notebook on GitHub: https://github.com/oncoray/UQ_OutcomeModelling.

### 4.1 Uncertainty quantification methods

There is no single metric for quantitatively expressing uncertainty, as uncertainty is problem-specific [18]. For regression tasks, measures of spread such as confidence intervals or standard deviations are typically used, while entropy or mutual information are considered for classification tasks. Other popular metrics rely on distances, e.g. Euclidean or Mahalanobis, in the input data domain or (simplified) latent representations thereof.

**Fig. 5:**
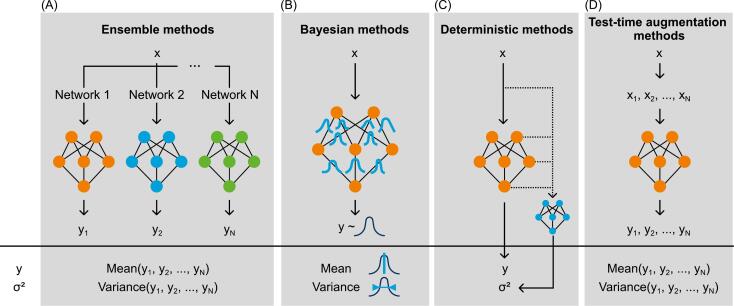
Methods for uncertainty quantification. Illustration of the basic principles for four different methods to quantify uncertainty using the example of a simple neural network. The methods can be used to model the relationship y = f(x) and to calculate a measure of uncertainty. Here, the variance σ^2^ is assessed. (A) Ensemble methods use several different networks. The prediction and a corresponding measure of uncertainty are calculated from the individual predictions. (B) Bayesian methods are based on Bayesian inference, resulting in a non-discrete model output. The resulting distribution of the outcome variable is used to calculate for instance the mean and variance. (C) Deterministic methods predict the outcome and their corresponding uncertainty simultaneously in a single-forward pass, e.g. using a connected second network in case of an external method. (D) In test-time augmentation methods, the input variable is modified resulting in several output variables. Graphical illustration after Gawlikowski et al. [18].

For simple models, e.g. based on logistic regression or Cox proportional hazards regression, uncertainty in predictions can be assessed using the underlying variance–covariance matrix of the fitted model coefficients or by bootstrapping [160]. Other models, including machine- and deep-learning-based approaches, may lack the required information to assess uncertainty directly or establish a complex non-linear mapping that prevents an analytical assessment of uncertainty. Therefore, methods have been developed to assess uncertainties in model predictions by other means [17,18,161,162]. Some of these methods also allow the separation of aleatoric and epistemic uncertainty, see Van Den Berg and Meliadò [163]. We describe common methods according to Gawlikowski et al. [18] below and summarise them in Table 2. An illustrative visualisation of the basic principles of the approaches is given in Fig. 5.

**Table 2:** Comparison of methods for uncertainty quantification.

	Ensemble methods	Bayesian methods	Deterministic methods	Test-time augmentation methods
Description	Multiple models are trained, each with different initialisations or input data samples. Uncertainty is aggregated from the diversity of predictions.	Models learn and infer distributions instead of point estimates. Approximations are used as the predictive distribution is intractable for high-dimensional networks.	Uncertainty is estimated in a single forward pass, either integrated in the model (internal) or as an extra component (external).	A single model is trained. Uncertainty is estimated by augmenting the input data leading to several different predictions.
Number of trained models	Multiple	1	1	1
Post-hoc implementation	No	Laplace approximation can be applied to already trained models; other methods need a dedicated architecture.	Only external methods can be applied to already trained models.	Yes
Possibility to distinguish epistemic and aleatoric uncertainty	Yes	Yes	No	No
Computational effort during training	High	High	Low	Low
Computational effort during inference	High	High	Low	High
Examples for applications in radiotherapy	[184,185]	[[186], [187], [188]]	[188,189]	[187,188,190]

**Ensemble methods** involve training multiple models that collectively form an ensemble [164,165]. Randomisation is used to induce variability. Models may be trained using different subsets of the data, i.e. different sets of samples or features [160], or random initialisations of model parameters such as weights of neural networks. This results in each model yielding a different prediction, with their aggregate reflecting uncertainty. While relatively straightforward to use and interpret, this method increases computational costs and cannot be used with existing models unless they are ensembles themselves. However, for some model architectures, e.g. neural networks, it may be possible to emulate ensemble predictions even from a single network using Monte Carlo dropout [166,167].

**Bayesian methods** follow the principle of Bayesian inference. In essence, instead of modelling and predicting single values and weights, models learn and infer distributions [168,169]. The predicted distribution directly contains probabilistic information for uncertainty estimation. Despite a strong theoretical background, this method has the problem that a prediction is practically intractable for today’s complex, multi-parametric models. For this reason, various approaches were developed to approximate the result like variational inference [170,171], Monte-Carlo-based sampling approaches [172,173], or Laplace approximation [168,174]. Monte Carlo dropout can also be interpreted as an approximation of Bayesian inference.

**Internal and external deterministic methods** estimate uncertainty in a single forward pass – unlike the previous methods. External methods train an additional model to predict uncertainty based on the input and output of the predictive model [175,176]. Internal methods are similar, but the uncertainty estimator is integrated within the architecture of the model. Dirichlet prior networks [177] and evidential neural networks [178] are examples of model architectures with internal deterministic methods.

Ensemble, Bayesian and deterministic methods cannot universally be used post-hoc – they need to be specifically supported at training time or with the help of an additional model. This limitation does not apply for **test-time augmentation methods**, which are model-agnostic because they work by sampling the data domain [179,180] and are a direct continuation of existing sensitivity analysis methods [181]. Test-time augmentation methods work by attempting to synthesise new realistic and somewhat similar samples in the data domain. For example, this method was used to assess how uncertainty in input data affects TCP and NTCP models [182,183]. Since the augmentation affects the input data and not the model, only aleatoric uncertainty can be inferred.

### 4.2 Applications

**Fig. 6:**
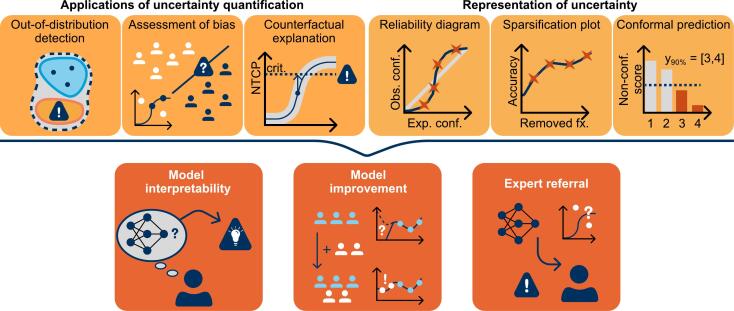
Use and representation of uncertainty quantification. Examples for applications and uncertainty representation methods are shown in the top row. These methods can be used to increase model interpretability, to address subpar model performance or to raise the need for expert referral, as illustrated in the bottom row. Abbreviations: NTCP: Normal tissue complication probability, crit.: critical value, Exp. conf.: Expected confidence, Obs. conf.: Observed confidence, fx.: fraction, Non-conf.: Non-conformity, y_90%_: 90% confidence prediction set.

Uncertainty quantification provides a tool to measure the reliability of a prediction. This information can be used in different tasks: (i) The uncertainty estimate can be used as a way for increasing interpretability and confidence in a decision. Especially for more complex models, non-reliable predictions are a critical problem that currently prevents clinical application. For example, the study by McGrath et al. [191] shows that uncertainty has an influence on whether model users trust in a model decision or not. (ii) Uncertainty estimates can be used to improve existing models. For example, uncertainty can draw attention to unconsidered or underrepresented subgroups or indicate that a domain shift happened. (iii) For models used in automated processes, e.g. contouring organs-at-risk in online-adaptive workflows, an uncertainty measure can be used to trigger referral to an expert. These main tasks can be addressed by various methods and representations, see Fig. 6.

Since models typically produce output regardless of the validity of the input data, uncertainty can be used to flag predictions for data that do not match the training data, i.e. due to being out-of-domain or having underwent a domain shift, see Section 2. This is called **out-of-distribution** (OOD) or anomaly detection [192,193]. In addition to the use of uncertainty estimates as a surrogate for OOD detection, there are other supervised and unsupervised approaches [192]. Supervised approaches intentionally include a sample of data anomalies or augmented data in the training process and learn to identify them [[194], [195], [196]]. Unsupervised approaches are for example auto-encoders [197,198] that learn to reconstruct the input data based on the output and label data as OOD if the reconstruction error exceeds a defined threshold.

Uncertainty quantification may also identify if and which **biases** between patient groups (see Table 1) exist in a model [199]. Assessing the presence of biases allows for addressing them accordingly to ensure that a model can generalise well. Uncertainty is moreover used in creating **counterfactual explanations**, i.e. to assess the smallest change in the input that would change a prediction [200,201]. Counterfactual explanations give insight in model decisions, the influence of features and about the robustness of a model. As an example, a model predicting chronic xerostomia after radiotherapy might be applied during treatment planning. Counterfactual explanation may indicate chronic xerostomia can be prevented by decreasing dose to salivary glands.

Uncertainty can be evaluated and presented at the global (cohort) level. A **reliability diagram** is a calibration plot that shows how well the uncertainty estimate is calibrated [[202], [203], [204]] through plotting the expected confidence based on the uncertainty estimate against the observed confidence. To construct a reliability diagram, an additional calibration dataset is used. For these data points, the confidence in the prediction is determined based on the uncertainty estimation. These are sorted into bins based on the confidence level. Now it is observed if the percentage of correct predictions in each bin resembles the expected confidence level. As an example for a classification task, in a bin with a confidence level of 0.6, it would be expected that 6 out of 10 predictions are correct. A well-calibrated model should result in a curve close to the diagonal, i.e. expected and observed confidence are balanced. A detailed explanation of how to create a reliability diagram for a regression and classification problem can be found in Nemani et al. [161].

The influence of uncertain predictions on model accuracy can also be shown by **sparsification plots** [205]. Such plots are created by iteratively removing the most uncertain predictions and assessing model accuracy at each step. Sparsification plots may help to determine thresholds for differentiating reliable and unreliable predictions. This is also useful to assess the impact of learning methods, which include the option to reject samples [206] by evaluating how sample rejection affects the model accuracy.

At the individual (patient) level, **conformal prediction** has gained popularity as an intuitive framework for uncertainty quantification [[207], [208], [209], [210]]. Conformal prediction constructs statistically rigorous prediction regions that ensure the inclusion of the true outcome with a user-specified probability. These regions are defined using a non-conformity measure, which quantifies the error or the extent to which a new data point differs from the existing dataset, or a dedicated calibration set. Then, based on the chosen confidence level, a threshold is established, allowing predictions with non-conformity scores below this threshold to be included within the prediction region. For classification tasks, this framework generates prediction sets containing all class labels necessary to ensure the specified confidence level, while for regression tasks, it produces prediction intervals. Thus, larger prediction regions correspond directly to higher levels of uncertainty. Conformal prediction has additional advantages. It is model-agnostic and can be seamlessly applied to any predictive model, including pre-trained ones, using a calibration dataset. Furthermore, conformal prediction is distribution-free, meaning it does not rely on prior assumptions about the underlying data distribution.

## 5 Discussion

In this review, we address causes and quantification methods for uncertainty pertaining to outcome modelling in radiation oncology. We illustrate how uncertainty is defined and where it originates, namely input and outcome data domains and the mapping established between these domains using models. We outline several methods for quantifying uncertainty and how this information can be used to enable a reliable and valid application of outcome models with transparent decision making in clinical practice.

Shifts and gaps in the data domain between training and application are major sources of model uncertainties. Comprehensive and bias-free data reduce uncertainty and help to create generalisable models. As shown in Section 3, the reasons for errors and bias in data are multifactorial. A data analysis plan systematically details and addresses key questions and is of central importance for reliable datasets. However, some issues are best resolved at multi-institutional or global scales. Large and clinically representative datasets need to be collected and must be made available [3,9]. In this context, interdisciplinary collaboration [152,211], international standards [212] and harmonisation are of crucial importance in order to ensure high data quality.

Errors and uncertainties may be reduced through AI-based tools in the data collection process, e.g. for plausibility checks, automated data extraction and sorting, as well as annotation and segmentation of medical images. These tools may reduce inter-observer variability, ensure better adherence to naming conventions and decrease errors made during manual data collection, among others. However, efforts in harmonisation and standardisation are also needed for the application of these methods themselves in order to avoid replacing inter-observer by inter-model variabilities. Consensus on methods and metrics is needed to ensure validation and harmonised clinical implementation, e.g. as highlighted for automatic segmentation tools [213,214].

Uncertainty in model decisions cannot be completely prevented. Hence, uncertainty quantification methods should be used to facilitate clinical translation. Furthermore, there is emerging need for the development and implementation of quality assurance methods for AI-based applications and tools [215,216] which can be addressed using uncertainty quantification, e.g. by allowing for detecting and reacting to domain shifts caused by technical innovation. However, despite general consensus on the importance of uncertainty quantification methods [163], they are rarely used for outcome models in radiation oncology [217]. Wahid et al. [217] showed that uncertainty quantification models were mostly used in tasks related to medical imaging like contouring, image reconstruction or synthesis. Another notable finding was that no studies explored the impact of uncertainty on end-user decision making, despite this often being a key motivating factor behind uncertainty quantification methods.

Uptake of uncertainty quantification methods may be affected by the current lack of standard “best” approaches for various tasks, e.g. for OOD detection [192]. The diversity of methods also causes uncertainty to be represented in different ways, which hampers comparison and limits interpretability. In this context, conformal predictions can be important, since they allow for presenting uncertainty in a consistent, interpretable manner [208].

Uncertainty quantification methods can support a reliable clinical integration of complex AI-based models [17]. However, for outcome prediction in radiation oncology, uncertainty quantification can be equally important for simple models, e.g. based on logistic regression, to enable a reliable translation and application into clinical practice. As some of these models are already being used clinically, e.g. for NTCP prediction [2,14], post-hoc application of uncertainty quantification can be applied. This also enables a fair comparison with complex approaches that are directly developed with an integrated uncertainty quantification and can allow for comprehensively assessing the added benefit of complex models.

A limitation of this topical review is the lack of a systematic literature research. Our aim was to present a general overview on uncertainty for outcome modelling in radiation oncology, its causes, and methods to estimate and address them. Consequently, we do not claim completeness and the presented concepts and methods should be interpreted as inspiration for application and further research.

To conclude, this topical review introduces fundamental concepts and existing methods for uncertainty quantification within the context of outcome modelling in radiation oncology. Because every model is influenced by uncertainties, both model developers and users should be aware of sources of errors, bias and other types of uncertainties and should address them accordingly to ensure accurate, reliable and transparent outcome predictions.

## Funding source

We acknowledge funding by the Deutsche Forschungsgemeinschaft (DFG, German Research Foundation) – 515284204 / SPP 2177. The funding agency was not involved in conducting this review.

## Declaration of competing interest

The authors declare that they have no known competing financial interests or personal relationships that could have appeared to influence the work reported in this paper.

Alex Zwanenburg is an Editorial Board Member for this journal and was not involved in the editorial review or the decision to publish this article.
